# A potential new role for myofibroblasts in remodeling of sub-rupture fatigue tendon injuries by exercise

**DOI:** 10.1038/s41598-018-27196-5

**Published:** 2018-06-12

**Authors:** Rebecca Bell, N. Remi Gendron, Matthew Anderson, Evan L. Flatow, Nelly Andarawis-Puri

**Affiliations:** 1 Sibley School of Mechanical and Aerospace Engineering, Cornell University, Ithaca, NY USA; 2 Nancy E. and Peter C. Meinig School of Biomedical Engineering, Cornell University, Ithaca, NY USA; 3 Leni and Peter W. May Department of Orthopaedics, Icahn School of Medicine at Mount Sinai, New York, NY USA; 4 Hospital for Special Surgery, New York, NY USA

## Abstract

Tendons are ineffective at repairing sub-rupture fatigue injuries. Accordingly, we evaluated whether an exercise protocol that we have previously found to decrease structural damage kinks in fatigue damaged tendons, leads to improvement in mechanical properties. We hypothesized that exercise that promotes repair of fatigue damage will decrease apoptosis and increase the population of myofibroblasts. Rat patellar tendons underwent *in vivo* fatigue loading for 500 or 7200 cycles. Animals resumed cage activity for 2-weeks, then either remained cage active or began treadmill running until sacrifice at 4- or 10-weeks post-fatigue loading. Exercise following fatigue damage increased the stiffness back towards naïve levels, decreased apoptosis and increased the population of myofibroblasts. Next, proteins associated with inhibition of apoptosis (Collagen VI) or activation of myofibroblast (pSmad 2/3, fibrillin, integrin subunits αV and α5) were evaluated. Data suggests that collagen VI may not be integral to inhibition of apoptosis in this context. Exercise increased pSmad 2/3 and fibrillin in the insertion region for the 7200-cycles group. In addition, exercise decreased integrin αV and increased integrin α5 in fatigue damaged tendons. Data suggests that a decrease in apoptosis and an increase in population of myofibroblasts may be integral to remodeling of fatigue damaged tendons.

## Introduction

Tendinopathy is a common musculoskeletal disease that affects the entire spectrum of society. While the underlying pathogenesis is not well understood, degenerative changes in ruptured tendons implicate a continuous process of accumulation of sub-rupture damage that ultimately leads to rupture^1–3^. After onset of rupture, the success of surgical management of ruptured tendons is contingent on the severity of existing degeneration^4^. Thus, establishment of non-surgical therapeutic interventions that could halt or reverse the accumulated damage would significantly improve patient care and diminish the need for surgical intervention.

Exercise, or physiological loading, is commonly prescribed for non-surgical management of tendinopathic tendons^5,6^. Given the vast patient-specific differences in severity of degeneration and time from onset of injury, it is not surprising that exercise has been clinically shown to lead to highly disparate outcomes^6,7^. Interestingly, using our previously established *in vivo* rat model of sub-rupture fatigue damage accumulation, we have shown similar disparate outcomes as clinical data, with further degeneration resulting from exercise that is initiated 1-day after fatigue loading but repair resulting from exercise that is initiated 2-weeks after fatigue loading^8^. Using a controlled animal model to determine the environment that is associated with exercise that effectively leads to repair will provide insight into the underlying mechanisms and identify therapeutic targets that can be modulated using biological approaches.

We have previously shown that one bout of moderate level fatigue loading leads to a 20% stiffness loss that is not recovered out to at least 6-weeks^9^. This lack of repair is accompanied by an early increase in cell death, or apoptosis, suggesting that a decrease in the population of cells that can repair the induced fatigue injury may account for the compromised capacity of fatigue damaged tendons to repair^10^. In contrast, we have shown that remodeling of fatigue damaged tendons from exercise is characterized by improved structure with decreased fatigue damage kinks and a decrease in proteins that are associated with tendinopathy, such as aggrecan and type III collagen^8^. Consequently, our overarching hypothesis is that exercise effectively leads to remodeling of fatigue damaged tendons by (A) inhibiting apoptosis thereby leading to an increase in the number of cells that can repair the induced damage, and (B) increasing the population of cells that can re-tension the damaged matrix. As a first step towards evaluating this hypothesis, the first objective of this study was to evaluate whether the previously observed decrease in damage kinks that results from exercise of fatigue damaged tendons is associated with a decrease in apoptosis and an improvement in mechanical properties. In addition, since myofibroblasts have been shown to play a critical role in contracture of wound healing, most typically through TGFβ pathways, we expect that they may play a similar role in re-tensioning the damaged matrix, albeit in this entirely distinct context^11,12^. This notion is further corroborated by studies suggesting that myofibroblasts may contribute to crimp formation in healthy tendons^13^. Consequently, the second objective of this study was to evaluate several proteins that are implicated in the modulation of apoptosis or the population of myofibroblasts to identify potential underlying mechanisms. More specifically, we hypothesize that exercise modulates apoptosis in fatigue damaged tendons by increasing type VI collagen, a pericellular matrix (PCM) component, and thereby stabilizing the mechanical micro-environment of the cells in damaged matrix. Studies in cartilage have suggested a protective role for type VI collagen against apoptosis, we expect that it may play a similar role in tendon response to therapeutic exercise^14,15^.

Similarly, we hypothesize that exercise modulates the population of myofibroblasts through modulation of integrin-based interactions with the matrix, namely integrin αV and α5, and through TGFβ pathways.

## Results

**Figure 1:**
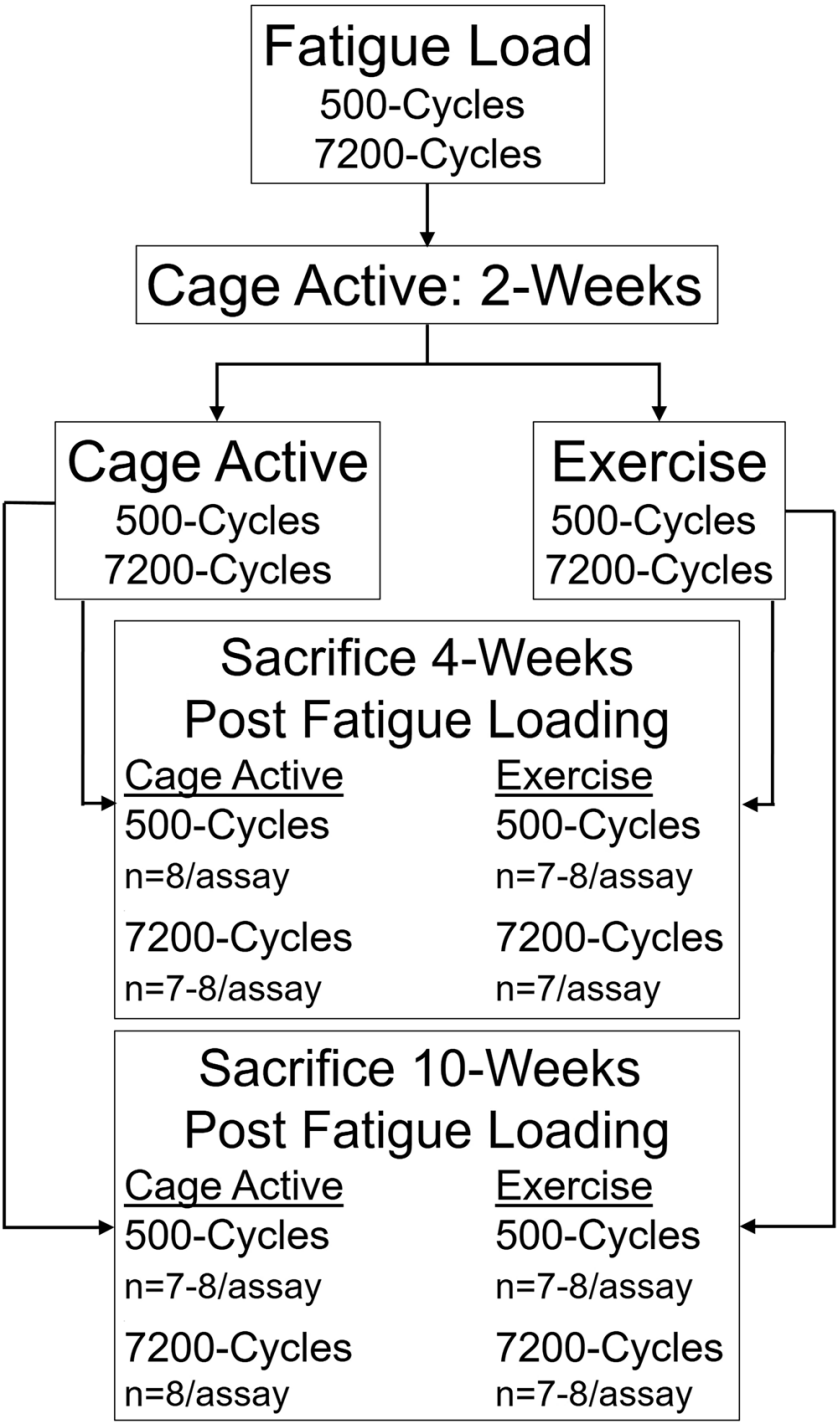
Schematic of study design.

First, we evaluated whether an exercise protocol that we have previously found to decrease structural damage kinks in fatigue damaged tendons, leads to improvement in mechanical properties. Per our previously established methods, rat patellar tendons underwent *in vivo* fatigue loading for 500 or 7200 cycles^10,16–18^. After fatigue loading, animals resumed cage activity for 2-weeks. After 2-weeks, rats either remained cage active or began treadmill running until sacrifice at 4- or 10-weeks post-fatigue loading (Fig. 1). As expected, analysis of the damage parameters confirmed that fatigue loading induced similar amounts of damage for the cage and exercise groups for each of the fatigue loading cycle groups (Supplemental Fig. 1). Consequently, any observed differences between the cage and exercise groups solely reflect the effect of exercise on fatigue loaded tendons.

### Mechanical Assessment

**Figure 2:**
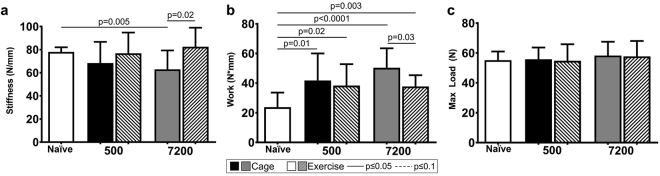
(**a**) 7200-cycles group of fatigue loading caused a 19% stiffness reduction at 10-weeks. Exercise restored the stiffness loss back to naïve levels. (**b**) Fatigue loading caused an increase of work for all groups. Exercise reduced work to maximum load for the 7200-cycles group. (**c**) Fatigue loading caused no change in maximum load. Data are shown as mean ± SD.

At 10-weeks post fatigue loading, animals were sacrificed and the patella-patellar tendon- tibia were harvested. Tendons were loaded to failure and stiffness, work to maximum load, and maximum load was determined. As expected based on previous findings^9^, at 10-weeks, tendons that were fatigue loaded to 7200-cycles exhibited a ~20% decrease (p = 0.02) in stiffness in comparison to naïve controls^17^ (Fig. 2a). Fatigue loading to 500 or 7200 cycles caused an increase in work to maximum load compared to naïve for both the exercise and cage groups (Fig. 2b). However, exercise increased the stiffness (p = 0.02) and decreased the work to maximum load (p = 0.03) of the 7200-cycles fatigue damaged tendons back towards naïve levels (Figs 1b and 2b, respectively). Fatigue loading to 500-cycles led to a non-significant decrease in stiffness and increase in work to maximum load, that was also modulated back towards naïve levels by exercise. Surprisingly, fatigue loading did not affect maximum load.

### Evaluation of proteins associated with apoptosis and activation of myofibroblasts

To generate new hypotheses regarding underlying mechanisms, proteins associated with inhibition of apoptosis (collagen VI) or activation of myofibroblast (pSmad 2/3, fibrillin, integrin subunits αV and α5) were evaluated.

### Evaluation of Apoptosis and Collagen VI

**Figure 3:**
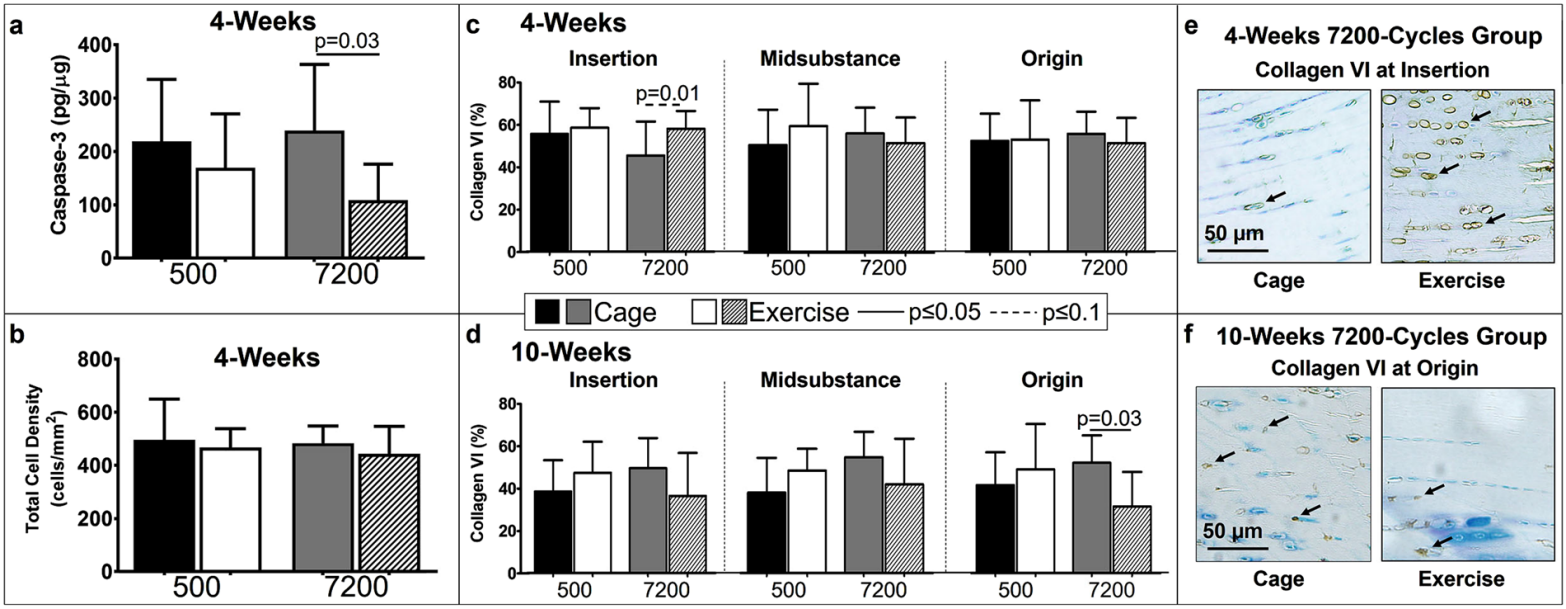
(**a**) At 4-weeks post fatigue loading, exercised decreased caspase-3 in 7200-cycles fatigue damaged tendons. (**b**)There was no difference in the total cell density for cage and exercise. (**c**) At 4-weeks post fatigue loading, exercise increased collagen VI at the insertion site for the 7200-cycles group. (**d**) At 10-weeks post fatigue loading, exercise significantly decreased collagen VI at the origin for the 7200-cycles group. Representative collagen VI staining showing (**e**) an increase of positive (brown) cells in the exercise group at the insertion at 4-weeks post fatigue loading and (**f**) a decrease of positive cells in the exercise group at the origin at 10-weeks post fatigue loading. Arrows indicate positive staining. Data are shown as mean ± SD.

At 4-weeks post fatigue loading, caspase-3 protein level, indicative of apoptosis, was assessed with ELISA kits (My Biosource, San Diego, CA). As hypothesized, exercise post-fatigue loading led to a significant decrease (p = 0.03) in the amount of caspase-3 when normalized by total protein (Fig. 3a) in the 7200-cycles fatigue loaded group. Further analysis confirmed that this effect of exercise on apoptosis in fatigue damaged tendons was not driven by any changes in population of cells, for instance by stimulating proliferation, since there was no significant difference in total cell density between cage and exercise groups (Fig. 3b). Exercise post-fatigue loading led to no significant effect on caspase-3 in the 500-cycles fatigue loaded group (Fig. 3a).

Collagen VI is a pericellular matrix protein that was hypothesized to be increased in conjunction with the decrease in apoptosis in the exercise group due to its role in modulating the micromechanical environment of cells. Accordingly, immunohistochemical (IHC) analysis was conducted to determine the effect of exercise on collagen VI at both 4- and 10-weeks post fatigue loading. Contrary to our hypothesis, at 4-weeks, exercise did not increase collagen VI in any region for the 500-cycles group (Fig. 3c). However, exercise led to the expected increase in collagen VI at the insertion region only in the 7200-cycles group (p = 0.1).

Similarly, at 10-weeks, exercise did not increase collagen VI in any region in the 500-cycles or in the insertion and midsubstance in the 7200-cycles group. Surprisingly exercise led to a decrease in collagen VI in the 7200-cycles group (p = 0.03) in the origin (Fig. 3d).

Evaluation of a subset of contralateral limbs (n = 4) showed that exercise in the absence of fatigue loading led to a modest increase in collagen VI(p = 0.1) at the insertion region while exercise post fatigue loading led to a significant increase (p = 0.03) when compared to naïve tendons (Supplemental Fig. 2a).

### Evaluation of Myofibroblast Population and Associated Proteins

**Figure 4:**
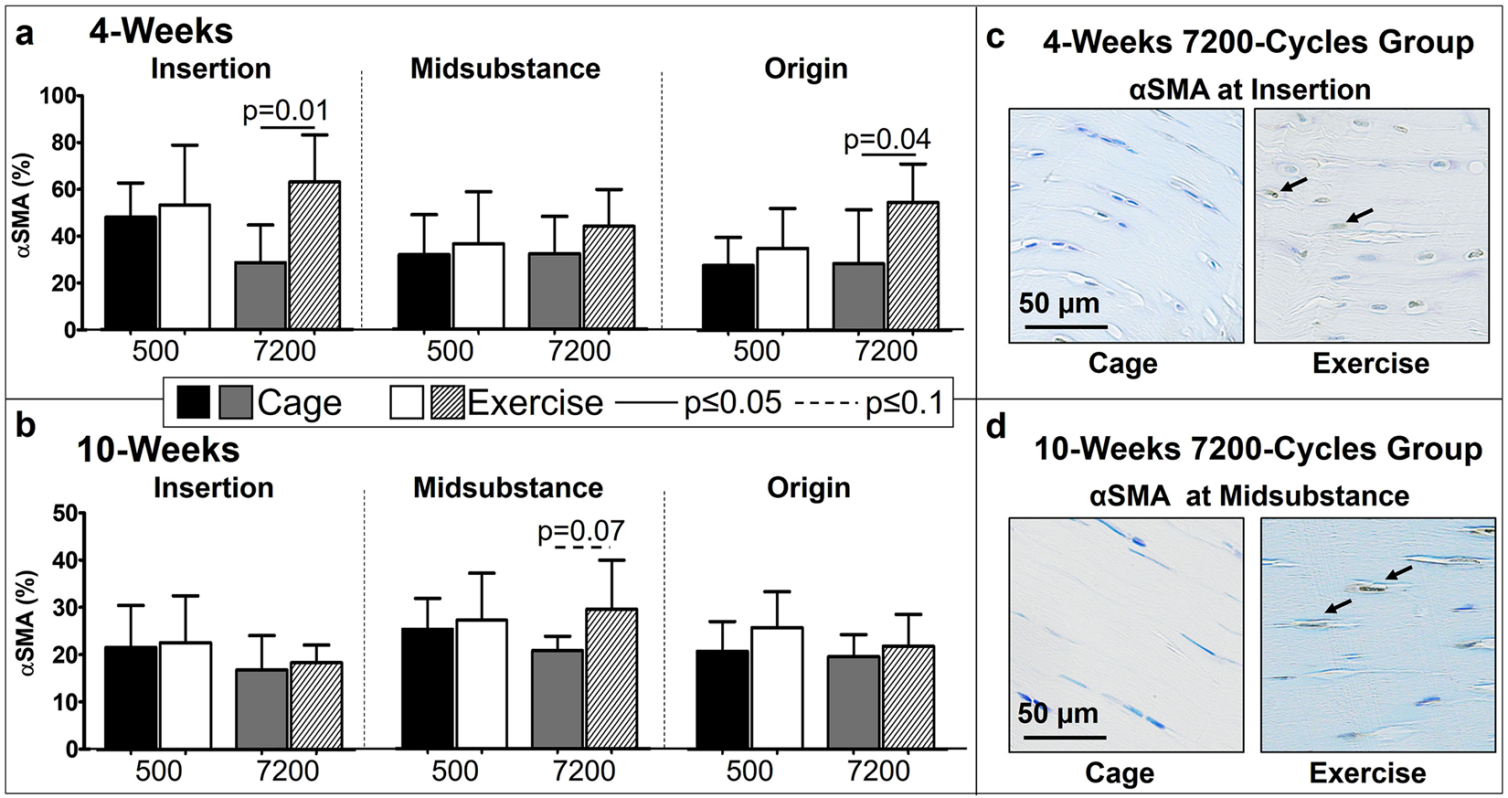
(**a**) At 4-weeks post fatigue loading, exercise significantly increased α-smooth muscle actin at the insertion for the 7200-cycle group. (**b**) At 10-weeks post fatigue loading, exercise increased α-smooth muscle actin at the midsubstance for the 7200-cycle group. Representative α-smooth muscle actin staining of the (**c**) insertion and (**d**) midsubstance showing an increase of positive (brown) cells in the exercise group. Arrows indicate positive staining. Data are shown as mean ± SD.

IHC analysis showed that, contrary to our hypothesis, exercise had no effect on the population of myofibroblasts for the 500-cycles group at 4- and 10-weeks post fatigue loading. As hypothesized, at 4-weeks, exercise increased the population of myofibroblasts in the 7200-cycles group at the insertion (p = 0.01) and origin (p = 0.04) (Fig. 4a). At 10-weeks, exercise increased the population of myofibroblasts in the midsubstance (p = 0.07) only for the 7200-cycles group (Fig. 4b). Further analysis confirmed that this effect of exercise on population of myofibroblasts was not driven by any changes in population of cells since there was no significant difference in total cell density between cage and exercise groups (Fig. 3b).

Evaluation of a subset (n = 4) of contralateral limbs showed that exercise in the absence of fatigue loading led to a significant increase in myofibroblasts at the insertion (p = 0.02), midsubstance (p = 0.01) and origin (p = 0.03) regions compared to naïve tendons. However exercise post fatigue loading led to a much greater significant increase in myofibroblasts at the insertion (p = 0.02) and origin (p = 0.02) when compared to exercise only (Supplemental Fig. 2b).

**Figure 5:**
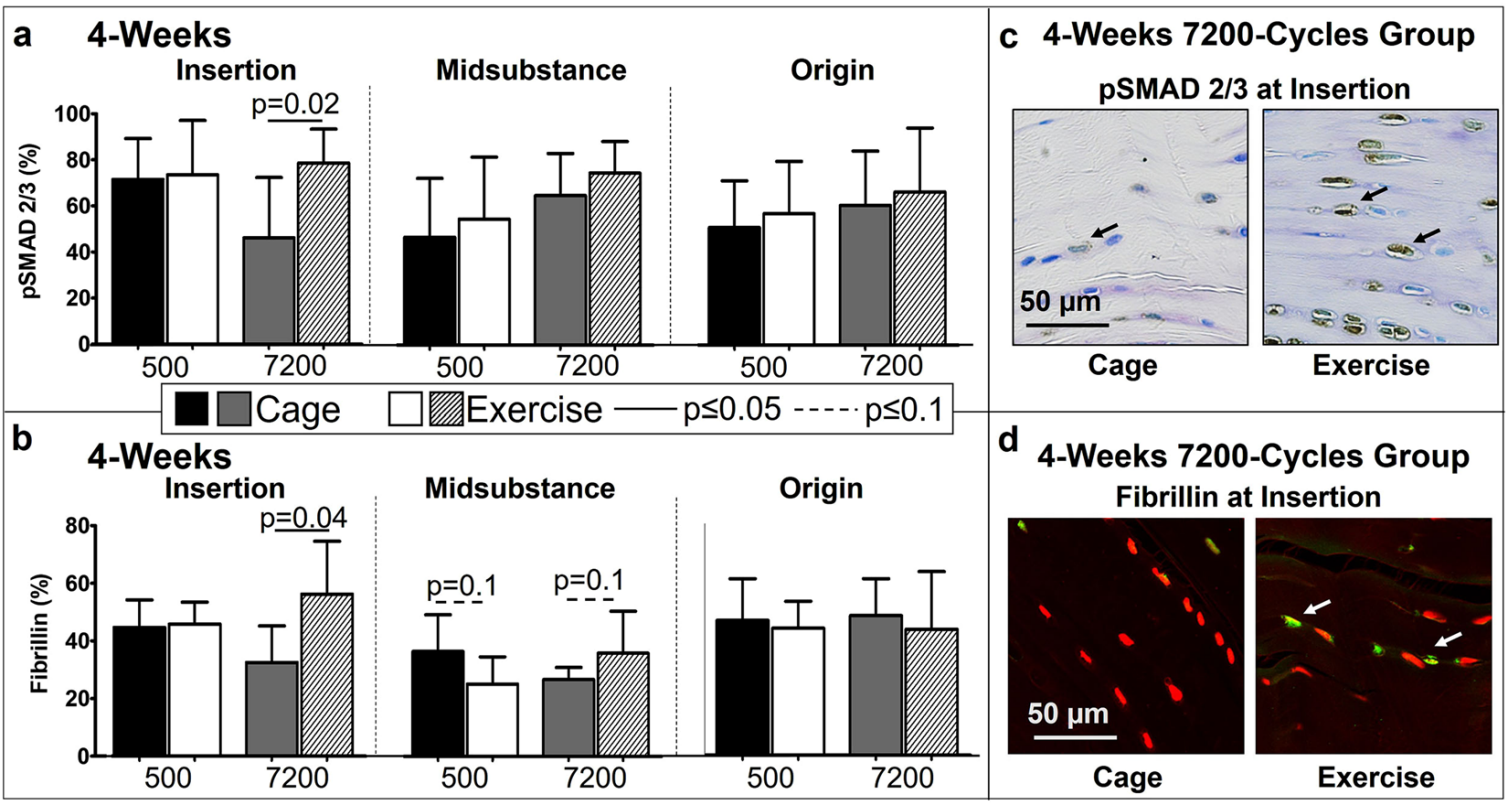
At 4-weeks post fatigue loading, exercise significantly increased (**a**) pSmad 2/3 and (**b**) fibrillin at the insertion for the 7200 cycle group. (**c**) Representative pSmad 2/3 staining of the insertion showing an increase of positive (brown) cells in the exercise group. (**d**) Representative fibrillin staining of the insertion showing an increase of positive (yellow/green) cells in the exercise group. Arrows indicate positive staining. Data are shown as mean ± SD.

**Figure 6:**
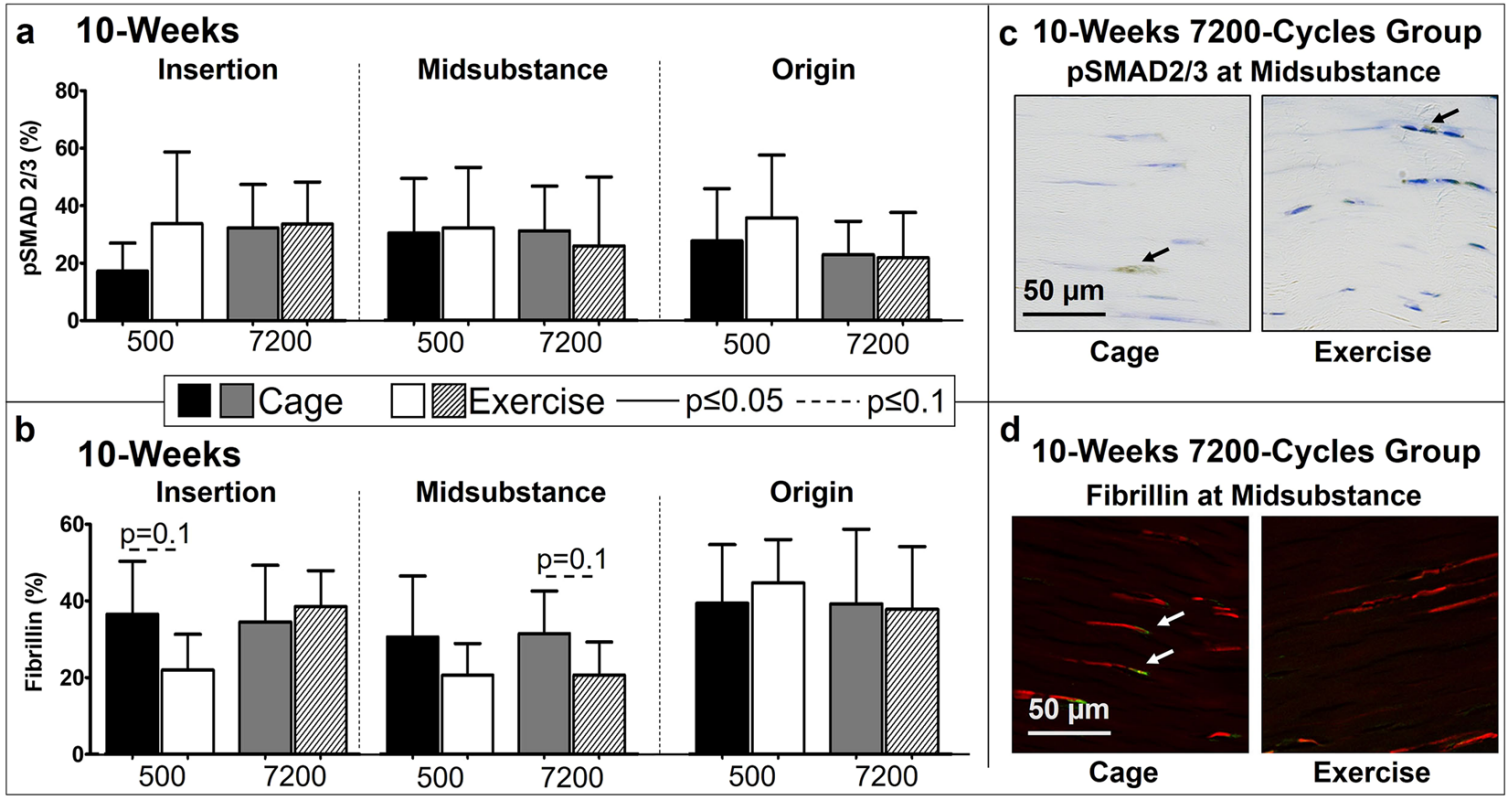
(**a**) At 10-weeks post fatigue loading, exercise had no effect on pSmad 2/3. (**b**) At 10-weeks post fatigue loading, exercise decreased fibrillin at the insertion for the 500-cycles group and at the midsubstance for the 7200-cycles group. (**c**) Representative pSmad 2/3 staining of the midsubstance showing no differences in percentage of positive (brown) cells. (**d**) Representative fibrillin staining at the midsubstance showing a decrease of positive (yellow/green) cells in the exercise group. Arrows indicate positive staining. Data are shown as mean ± SD.

Since TGFβ is commonly implicated in differentiation of myofibroblasts^19^, IHC analysis was conducted to evaluate the effect of exercise on the downstream phosphorylated Smad 2/3 (pSmad 2/3) and fibrillin-1, an ECM protein that sequesters TGFβ. Contrary to our hypothesis, exercise had no effect on pSmad 2/3 for the 500-cycles group for both the 4-week and 10-week groups (Figs 5a, 6a). Surprisingly, exercise decreased fibrillin in the 500-cycles group in the midsubstance at 4-weeks (p = 0.1, Fig. 5b) and in the insertion at 10-weeks (p = 0.1, Fig. 6b) post fatigue loading. As hypothesized, at 4-weeks, exercise increased pSmad 2/3 (p = 0.02, Fig. 5a) and fibrillin-1 (p = 0.04, Fig. 5b) in the insertion region only for the 7200-cycles group. Interestingly, in the midsubstance, exercise led to an increase in fibrillin-1 at 4-weeks (p = 0.1, Fig. 5b) but a decrease at 10-weeks (p = 0.1, Fig. 6b), suggesting that the observed decreases in fibrillin-1 could be in response to a potential negative feedback loop.

**Figure 7:**
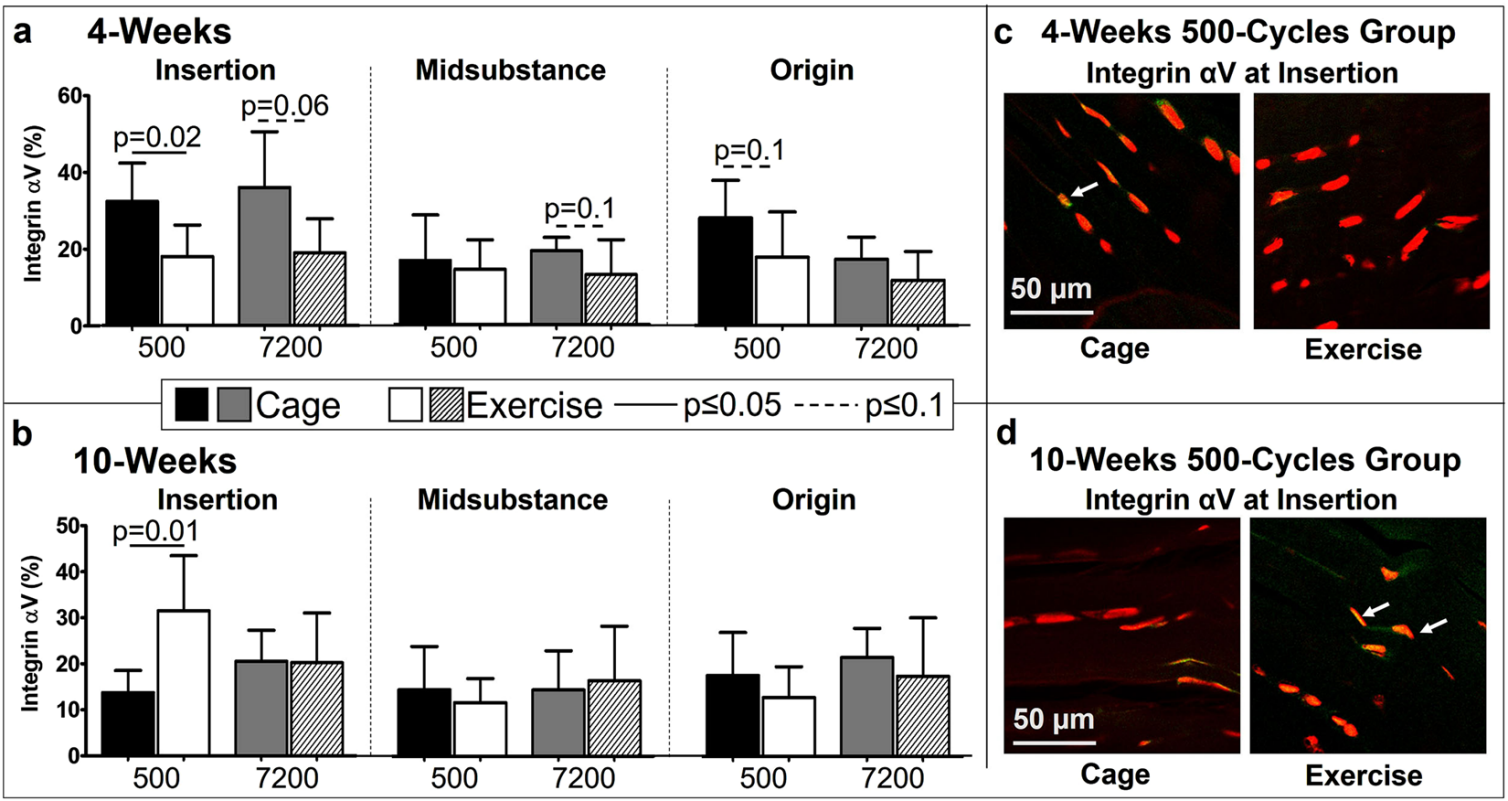
(**a**) At 4-weeks post fatigue loading, exercise decreased integrin αV at the insertion for the 500-cycles group. (**b**) At 10-weeks post fatigue loading, exercise increased integrin αV at the insertion for the 500-cycles group. **(c**) Representative integrin αV staining of the insertion showing a decrease in positive (yellow/green) cells in the exercise group at 4-weeks and at 10 weeks (**d**) showing an increase in positive (yellow/green) cells in the exercise group. Arrows indicate positive staining. Data are shown as mean ± SD.

Integrin subunits αV and α5 contribute to regulation of myofibroblast activation by TGFβ^11,20^. IHC analysis showed that exercise generally led to the expected decrease in the lower-force bearing integrin αV and increase in the higher-force-bearing integrin α5 in fatigue damaged tendons at 4-weeks. More specifically, at 4-weeks post fatigue, exercise decreased integrin αV for the 500- cycle group (p = 0.02) at the insertion and origin (p = 0.1) (Fig. 7a). Similarly, at 4-weeks post fatigue, exercise decreased integrin αV for the 7200-cycles group at the insertion (p = 0.06) and midsubstance (p = 0.1). Contrary to our hypothesis, at 10-weeks post fatigue, exercise increased integrin αV in the 500-cycles group at the insertion (p = 0.01) (Fig. 7b). There was no other effect of exercise on integrin αV on fatigue damaged tendons at 10-weeks.

**Figure 8:**
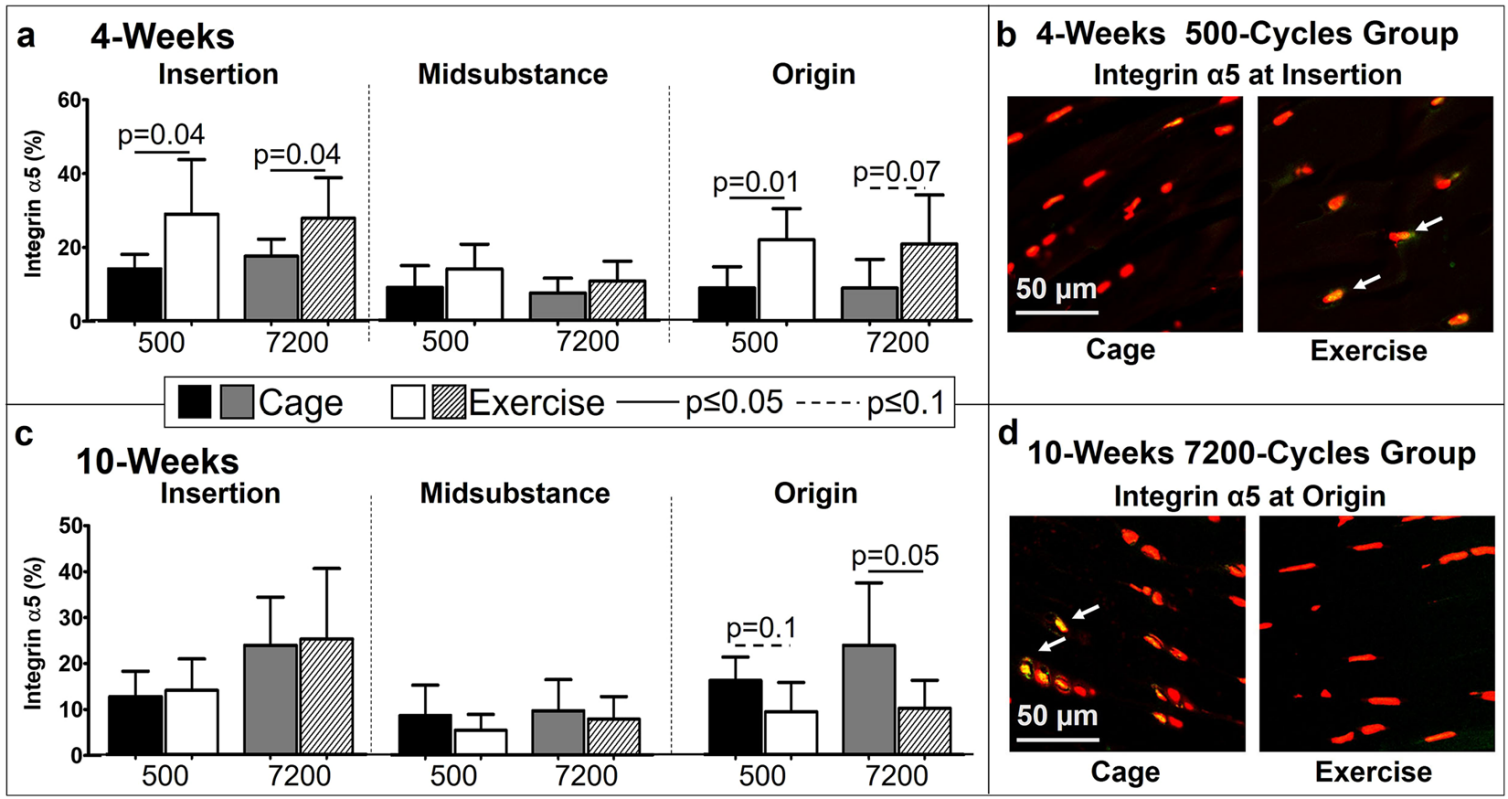
(**a**) At 4-weeks post fatigue loading, exercise significantly increased integrin α5 at the insertion for both the 500- and 7200-cycles groups. (**b**) At 10-weeks post fatigue loading, exercise significantly decreased integrin α5 at the origin for the 7200-cycles group. (**c**) Representative integrin α5 staining of the insertion showing an increase in positive (yellow/green) cells in the exercise groups at 4-weeks. (**d**) Representative integrin α5 staining of the origin showing a decrease in positive (yellow/green) cells in the exercise groups at 10-weeks. Arrows indicate positive staining. Data are shown as mean ± SD.

As expected at 4-weeks post fatigue, exercise led to an increase in integrin α5 at the insertion and origin in both the 500-cycles (p = 0.04 and p = 0.01, respectively) and 7200-cycles (p = 0.04 and p = 0.07, respectively) (Fig. 8a). Contrary to our hypothesis, at 10-weeks post fatigue, exercise led to a decrease in integrin α5 at the origin for both the 500-cycles (p = 0.1) and 7200-cycles (p = 0.05) groups (Fig. 8b). There was no other effect of exercise on integrin α5 on fatigue damaged tendons.

## Discussion

We have previously shown that 1-bout of moderate level fatigue loading (7200-cycles) results in an immediate and sustained 20% stiffness loss^9^, suggesting that tendons do not effectively repair accumulated sub-rupture fatigue damage. Consequently, we have begun to evaluate the utility of physiological loading to promote remodeling in fatigue damaged tendons. Our previous findings have shown that the timing of initiation of exercise after onset of sub-rupture injury is critical in driving remodeling versus further degeneration in structural matrix damage kinks^8^. In this study, we first confirmed that the reduction in structural damage kinks from exercise leads to restoration of the stiffness of the damaged tendon.

An increase in apoptosis has been clinically associated with tendon degeneration and rupture. For instance, torn supraspinatus tendons exhibit significantly higher apoptotic activity in lesion edges than in intact tendons^21–23^. Apoptotic activity has also been shown to increase in patellar tendons that exhibit painful chronic tendinopathy^24^. We have previously shown that apoptosis is increased early in the pathogenesis of tendinopathy, with marked increases occurring within 1-week after 1-bout of moderate level fatigue loading^10^. Accordingly, we have previously hypothesized that the early increase in apoptosis may be a significant contributing factor to the impaired capacity of fatigue-damaged tendon to repair. In this study, our data supports our new hypothesis that exercise that leads to effective repair of fatigue damaged tendons leads to inhibition of apoptosis.

The mechanisms that drive increase apoptosis in fatigue damaged tendons, and conversely its decrease with exercise, remain unknown. We expect that cells in regions of high severity of ECM damage may experience loss of interactions with the ECM and diminished loading that leads to loss of homeostatic tension. Interestingly, *in vitro* studies have shown that apoptosis can be induced in tendon cells from loss of homeostatic tension^25^. Accordingly, we expected that increase in the PCM protein, collagen VI, could be a mechanism by which cells in regions of ECM damage modulate their micromechanical environment and restore homeostatic tension. Studies in cartilage further corroborate this potential structural role of collagen VI in preventing apoptosis^26^, suggesting that it may play a similar role in tendon. However, contrary to our hypothesis, exercise led to a small increase in collagen VI and only in the insertion site of fatigue damaged tendons. Since apoptosis is initiated within the first week after induction of fatigue damage injury^10^, collagen VI may play a more prominent role earlier than at the 4-week and 10-weeks timepoints evaluated. Alternatively, collagen VI may be a prominent PCM component in tissues that commonly bear compressive loads but not tensile load bearing tissues, suggesting that alternative PCM proteins should be evaluated in the tensile load bearing tendon.

Our previous studies have shown that exercise promotes remodeling of sub-rupture fatigue damage by decreasing damage kinks^8^. Since myofibroblasts are capable of tensioning their surrounding matrix^27^ and have been previously shown to be increased by exercise in healthy tendons^28^, we hypothesized that myofibroblasts, which are not typically associated with subrupture injury, may play an essential role in remodeling of fatigue damaged tendons. Our results supported our hypothesis, showing that exercise that ultimately leads to remodeling of fatigue damaged tendons, also leads to an increase in population of myofibroblasts. More specifically, the population of myofibroblasts was particularly increased at the insertion and origin, which are the regions that have been previously shown to accumulate the greatest amount of damage^8,10^. Our results suggest a novel role for myofibroblasts in remodeling of sub-rupture fatigue damage tendon injuries.

Multiple pathways have been implicated in activation and differentiation of myofibroblasts^29^. As expected, activation of TGFβ, as indicated by pSMAD 2/3, correlated with increase in myofibroblasts at the insertion site. Interestingly, fibrillin-1, an ECM protein that sequesters TGFβ, was also increased in this region, suggesting that fibrillin-1 may be highly implicated in modulation of TGFβ and myofibroblasts activation in remodeling of fatigue damaged tendons. In addition, integrins αV and α5 are implicated in activation of myofibroblasts by TGFβ^30^. Interestingly, we found that regions that exhibit an increase in myofibroblasts, also exhibit an increase in integrin α5, suggesting a critical role for integrin α5 in remodeling of fatigue damage through activation of myofibroblasts. However, the increase in integrin α5 that was also found in groups that exhibited a decrease in damage without an increase in myofibroblasts suggest an alternative role of integrin α5 in remodeling of damage kinks by increasing cell contractility^31,32^.

We have previously shown that exercise that is initiated 2-weeks post fatigue loading injury leads to remodeling of damage kinks^8^, and have now shown an associated recovery in stiffness loss. Our previous work has led us to generate a hypothesis implicating a decrease in apoptosis and increase in myofibroblasts in remodeling of fatigue damaged tendons by exercise. This study, represents a first step in interrogating mechanisms that underlie effective remodeling of fatigue damaged tendons by identifying correlated changes in apoptosis, myofibroblasts, and associated inhibitors and activators, respectively. Future studies will build on this data to utilize biological inhibitors of apoptosis and activators of myofibroblasts to directly determine their role in remodeling of fatigue damaged tendons. Another limitation to this study that exercise protocols that are developed in animal models cannot be directly translated as a therapeutic for human injuries. However, utilizing a well-controlled method for inducing sub-rupture fatigue injury along with a well-controlled exercise protocol is optimal for determining the mechanism by which sub-rupture injuries can be prompted to remodel. Thus, our studies will effectively identify potential biological therapeutics that can be translated to manage tendon sub-rupture tendon injuries humans.

Our previous studies have generally indicated that tendons are highly ineffective at repairing sub-rupture injuries^8,9,10,33^. Furthermore, sub-rupture injuries promote apoptosis and a temporal biological response that is further muted in correlation with the severity of induced damage^10^. Since exercise can lead to remodeling of healthy tendons^34^, we subsequently evaluated its utility to promote repair in sub-rupture fatigue damaged tendons. As expected, our previous findings indicated that exercise can promote further degeneration or prompt repair^8^; a highly disparate outcome that is associated with timing of initiation of exercise after injury. Thus, our previous finding showing that initiating exercise 2-weeks after fatigue loading leads to repair of sub-rupture fatigue damage tendon injuries, suggested this model can serve as platform to interrogate the biological environment associated with effective remodeling. Our data provided evidence to support our overarching hypothesis, that a decrease in apoptosis and increase in myofibroblasts may be integral to promoting remodeling of fatigue damaged tendons. Future studies will interrogate the role of myofibroblasts and apoptosis in remodeling of fatigue damaged tendons.

## Methods

All experiments were approved and performed in accordance of Cornell University’s and Icahn School of Medicine at Mount Sinai’s Institutional Animal Care and Use Committees. 128 Sprague-Dawley 9–10 month old female retired breeder rats (Charles River Laboratories International, Inc., Wilmington, MA) were anesthetized and their left patellar tendons were fatigue loaded per our previously established protocol^8,9,10,33^. Briefly, the tibia was exposed, clamped and secured to a base. The patella was exposed, clamped and connected in series to a 50-lb load cell (Transducer Techniques, Temecula, CA) and to an Instron machine (Instron 8841 Materials Testing Solutions, Norwood, MA). The patellar tendon was fatigue loaded from 1 to 40 N at 1 Hz for either 500 or 7200 cycles. Diagnostic tests were performed pre and post fatigue loading and consisted of loading from 1 to 15 N at 1 Hz for 420 (pre) or 120 (post) cycles. As previously described, parameters that are indicative of the damage induced were calculated from diagnostic tests and were used to confirm that fatigue loading induced similar amounts of damage in all groups^9,10,33^. After fatigue loading, the skin incisions were sutured using prolene 6-0 sutures (Ethicon, Somerville, NJ) and buprenorphine (0.06 mg/ml/kg) was administered.

All animals resumed cage activity after fatigue loading for 2-weeks. At 2-weeks post-fatigue, group 1 (500-cycles) and group 3 (7200-cycles) continued cage activity. Group 2 (500-cycles) and group 4 (7200-cycles) began the exercise regimen that consisted of treadmill running for 30 min/day for 5 days/week at 17 m/min. The animals were sacrificed either 4- or 10-weeks post-fatigue loading (Fig. 1a).

### Mechanical Testing

Since others^35,36^ have shown long term exercise is needed to effect mechanical properties, 10-weeks was chosen as the only timepoint for mechanics. Animals (n = 8 per group) were sacrificed and the patella-patellar tendon- tibia were harvested and frozen at −20 °C. At the time of testing, the tibias were potted in PMMA bone cement. The potted tibia was secured to the base and the patella was clamped with the same clamp from the fatigue loading and attached in series to the load cell and Instron. Tendons were loaded with a 2 N preload and held for a minute before a pull-to-failure test was conducted at a rate of 0.3%/second. The tendons were kept moist with PBS throughout testing. Preliminary studies have shown that neither fatigue loading nor treadmill running result in a change in the cross sectional area of the tendon at 6-weeks, leading us to expect no effect at the 10-week timepoint evaluated in this study (Naïve- 3.7+/− 0.8 mm^2^, 6-weeks post-fatigue loaded- 3.9+/− 0.7 mm^2^, 6-week running- 3.8+/−0.7 mm^2^). Accordingly, only structural properties were calculated (stiffness, work to failure, and maximum load).

### Enzyme-Linked Immunosorbent Assay (ELISA)

Since our previous work has shown apoptosis peaks within the first two weeks of fatigue loading consistent with other tissues following injury^10,37,38^, caspase-3 was only measured at 4-week timepoint. Animals (n = 8 per group) were sacrificed and the patellar tendon was immediately harvested and flash frozen. The tendons were pulverized and incubated at 4 °C in 0.5 ml of acetic overnight. The supernatant was collected and the remaining precipitate was digested with 0.5 ml pepsin solution (0.1 mg/ml) for 24 hours. The pepsin digestion was repeated until all fragments were solubilized and the supernatant was collected after each step. The remaining un-solubilized fragments were digested with 0.5 ml of elastase solution (0.1 mg/ml). All supernatant was combined and neutralized with 1 M Tris base. Caspase-3 protein level was assessed with ELISA kits (My Biosource, San Diego, CA) and normalized to total protein. Total protein was assessed with Bradford Protein assay (Bio-Rad Laboratories, Hercules, CA).

### Immunohistochemistry (IHC)

At both 4- and 10-weeks post fatigue, animals (n = 6–8 per group per timepoint) were sacrificed and the quadriceps-patella-patellar tendon-tibia complex was harvested and fixed under 2 N of tension in Z-fix (Anatech Ltd, Battle Creek, MI) and decalcified (Decal Chemical Corporation, Tallman, NY). The complex was embedded in paraffin and sectioned at 6 µm. Proteinase-K was used for antigen retrieval and non-specific binding was blocked with Dako Protein block (Agilent, Santa Clara, CA). Staining for α-smooth muscle actin (αSMA, a myofibroblast marker, ab124964, 1:1000, Abcam, Cambridge, MA), phosphorylated Smad 2/3 (pSmad 2/3, sc11769, 1:500, Santa Cruz Biotech, Dallas, TX), type VI collagen (Col VI, 1:2000 Abcam, Cambridge, MA), integrin subunit αV (ab179475, 1:200, Abcam, Cambridge, MA), integrin subunit α5 (ab150361, 1:100, Abcam, Cambridge, MA), and fibrillin-1 (abin265420, 1:50, antibodies-online, Atlanta, GA) was conducted. For αSMA, pSmad2/3, Col VI, chromogen-based secondary with toluidine blue as a counter stain was used. For αV, α5, and fibrillin-1, alexa fluor 488 secondary with NucRed (Thermo Fisher, Waltham, MA) as a counter stain was used.

Images were acquired using a 20x lens at the insertion, midsubstance and origin. The origin and insertion regions were defined by creating a trapezoid with the tidemark of the fibrocartilage region of the enthesis being one border and the tendon being the other. For the midsubstance, 3 regions (400 µm by 400 µm) 1000–1500 microns apart were imaged. A counterstain (toluidine blue (chromogenic) or NucRed(fluorescent)) was used to highlight negative stained cells^8,10^. A blinded user counted positively and negatively stained cells for all chromogenic stains. Positively and negatively stained fluorescent cells were counted using a custom Matlab program. The percentage of positively stained cells, positive cell density (data not shown), and total cell density were subsequently determined.

### Statistical Analysis

All data was tested for normality using the Shapiro-Wilk test and were found to be normally distributed. For the mechanical testing, each group was compared to naïve using two-tailed Student’s test. For the remaining assays, the cage and exercise groups were compared using two-tailed Student’s t-test for each of the 500-cycles and 7200-cycles groups. Significance was set at p ≤ 0.05 denoted with “*” and a trend at p ≤ 0.1 denoted with “#”. All data is presented as mean ± standard deviation (SD).

### Data Availability

The datasets generated in this current study are available from the corresponding author on reasonable request.

## Electronic supplementary material

Supplementary Figure 1 and 2
