# Supplementary material for: A potential new role for myofibroblasts in remodeling of sub-rupture fatigue tendon injuries by exercise
Source: Sci Rep. 2018 Jun 12;8:8933. doi: 10.1038/s41598-018-27196-5 (PMC5997675; doi:10.1038/s41598-018-27196-5)
Supplement: Supplementary file 1 — Supplementary Figure 1 and 2 [file 41598_2018_27196_MOESM1_ESM.pdf]

A potential new role for myofibroblasts in remodeling of sub-rupture fatigue tendon injuries by exercise

Rebecca Bell, PhD[1], N. Remi Gendron[3], Matthew Anderson, MD[3], Evan L. Flatow, MD[3], Nelly Andarawis-Puri, PhD*[1,2,4]

[1] Sibley School of Mechanical and Aerospace Engineering

Cornell University, Ithaca, NY, USA

[2]Nancy E. and Peter C. Meinig School of Biomedical Engineering

Cornell University, Ithaca, NY, USA

[3]Leni and Peter W. May Department of Orthopaedics

Icahn School of Medicine at Mount Sinai, New York, NY,

[4] Hospital for Special Surgery

New York, NY, USA

Address correspondence to:
*Nelly Andarawis-Puri, PhD
Sibley School of Mechanical and Aerospace Engineering
Cornell University
Ithaca, NY 14850
Na424@cornell.edu

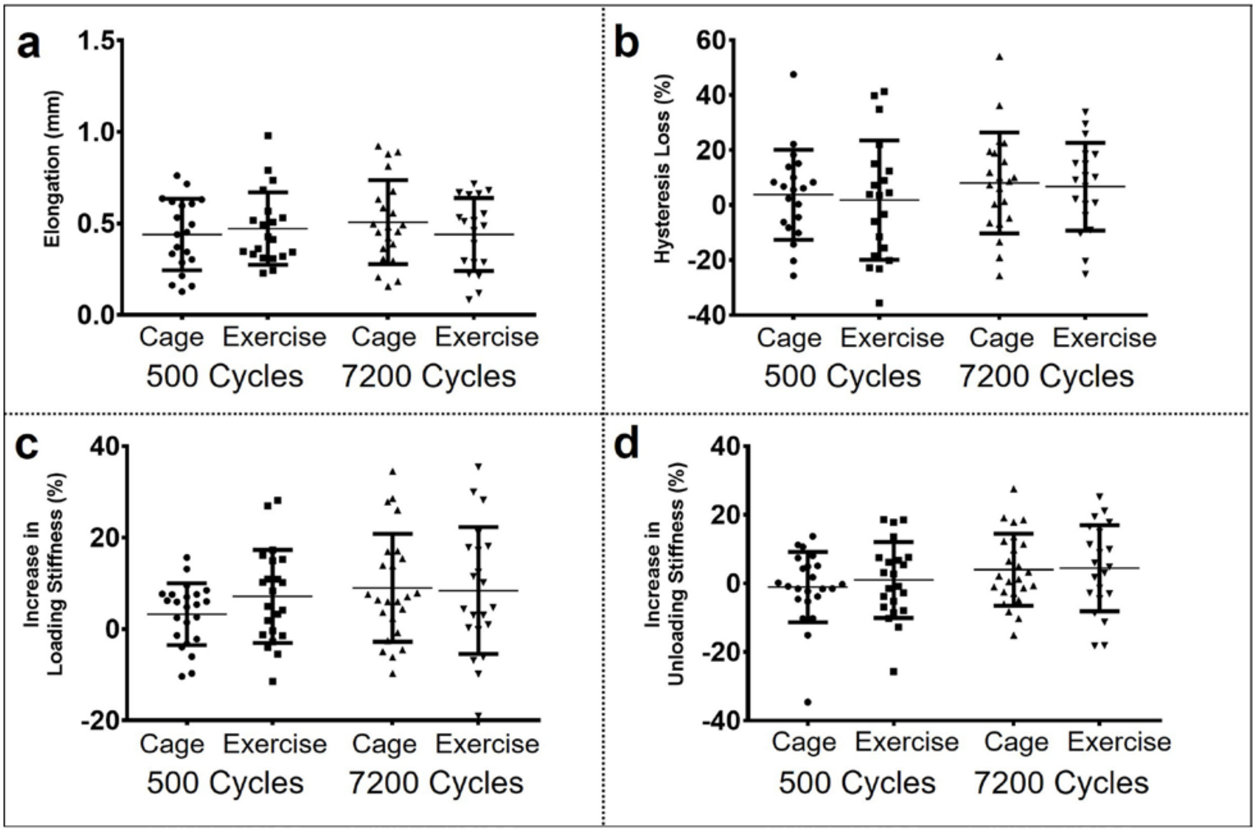

Supplementary Figure 1: (a) Fatigue loading induced similar amounts of damage indicated by (a) elongation, (b) hysteresis loss, (c) increase in loading stiffness, and (d) increase in unloading stiffness for both 500 cycles and both 7200 cycles groups.

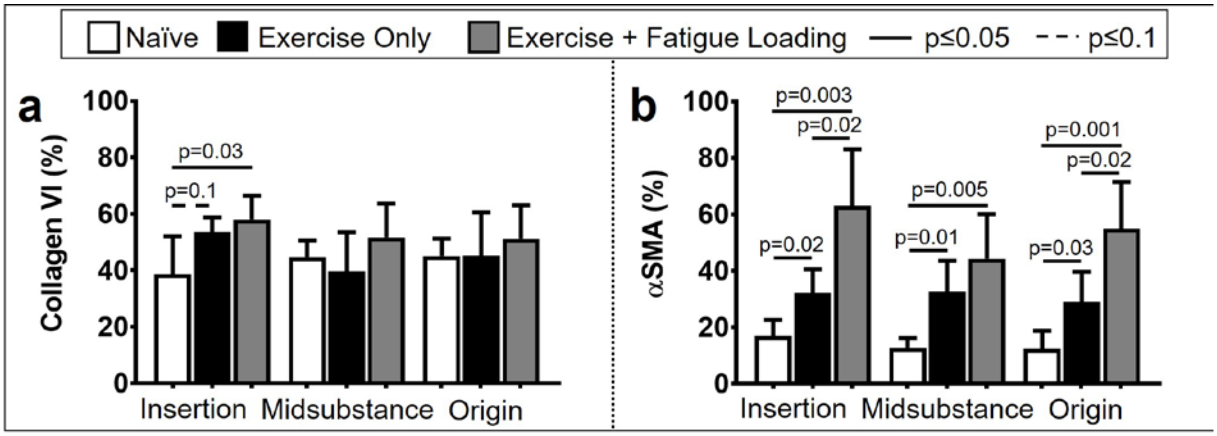

Supplementary Figure 2- a) Exercise post fatigue loading to 7200 cycles significantly increased collagen VI at the insertion. (b) Contralateral limbs that underwent exercise only exhibited a significant increase in α-smooth muscle actin in all regions compared to naïve tendons. However exercise post fatigue loading to 7200 cycles led to a significantly greater increase in α-smooth muscle actin in the insertion and origin compared to exercise alone. Data are shown as mean ± SD.
